# Focal cartilage defects in the knee –a randomized controlled trial comparing autologous chondrocyte implantation with arthroscopic debridement

**DOI:** 10.1186/s12891-016-0969-z

**Published:** 2016-03-08

**Authors:** Per-Henrik Randsborg, Jan Brinchmann, Sverre Løken, Heidi Andreassen Hanvold, Tommy Frøseth Aae, Asbjørn Årøen

**Affiliations:** Department of Orthopedic Surgery, Akershus University Hospital, Lørenskog, Norway; Department of Immunology and Norwegian Center for Stem Cell Research, Oslo University Hospital, Rikshospitalet, Oslo, Norway; Department of Orthopedics, Oslo University Hospital, Oslo, Norway; Department of Physiotherapy, Akershus University Hospital, Lørenskog, Norway; Department of Orthopedic Surgery, Kristiansund Hospital, Kristiansund, Norway; Institute of Clinical Medicine, University of Oslo, Oslo, Norway; Oslo Sports Trauma Research Centre, Norwegian School of Sport Sciences, Oslo, Norway

## Abstract

**Background:**

Focal cartilage injuries in the knee might have devastating effect due to the predisposition of early onset osteoarthritis. Various surgical treatment options are available, however no statistically significant differences have been found between the different surgical treatments. This supports the suggestion that the improvement might be a result of the post-operative rehabilitation rather than the surgery itself. Autologous chondrocyte implantation (ACI) has become a recognized treatment option for larger cartilage lesions in the knee. Although ACI has been compared to other surgical treatment such as microfracture and mosaicplasty, it has never been directly compared to simple arthroscopic debridement and rehabilitation alone. In this study we want to increase clinical and economic knowledge about autologous chondrocyte implantation compared to arthroscopic debridement and physical rehabilitation in the short and long run.

**Methods/Design:**

We will conduct a randomized controlled trial to compare ACI with simple arthroscopic debridement (AD) and physiotherapy for the treatment of cartilage lesions in the knee. The study will include a total of 82 patients, both men and non-pregnant women, with a full thickness cartilage defect in the weight bearing area of the femoral condyles or trochlea larger than 2 cm2. The lesion must be symptomatic, with a Lysholm score less than 75.

The two treatment groups will receive identical rehabilitation protocol according to a modification of Wondrasch et al., which is an active rehabilitation and education program divided into 3 phases: accommodation, rehabilitation and return to activity. The patients will be followed for 24 months, with additional late follow-ups at 5 and 10 years to monitor the potential onset of osteoarthtitis.

The primary outcome measure will be the difference in the KOOS knee-related quality of life (QoL) subscore in the ACI group compared to the AD group at 2 years. A combination of self-explanatory questionnaires, clinical parameters, clinical hop tests and radiographs and Magnetic Resonance Imaging (MRI) will be used as secondary endpoints.

**Discussion:**

This is the first study with a high level of evidence to compare ACI with simple debridement and physiotherapy for the treatment of isolated symptomatic full thickness lesions of the knee.

**Trial registration:**

ClinicalTrial NCT02636881 (21 December 2015)

## Background

The articular surfaces of joints are covered with hyaline cartilage, a unique tissue with extreme load shearing and low-friction properties. However, these characteristics come with a cost. It is aneural and avascular which explains its limited ability to self-repair [1–3]. Damage to the cartilage, which is common in young active adults, will therefore lead to permanent damage. These injuries are very common, with a reported prevalence of 12 % in the population [4]. Focal cartilage lesions predispose to development of early onset osteoarthritis, which in turn may lead to long rehabilitation periods and loss of function and time off work. Musculoskeletal problems are one of the major reasons for workers compensation and especially for younger patients this may lead to a significant reduced quality of life and loss of income [5–8]. Treatment of symptomatic cartilage injuries in the knee is therefore of particular interest and importance to the patient, the surgeon and the society [1, 9, 10].

The ideal treatment for isolated cartilage injuries aims at recreating a healthy hyaline-type tissue with similar strength and durability as the normal cartilage in the rest of the joint. Current surgical treatment options include debridement, microfracture, autologous osteochondral transplantation (mosaicplasty) and autologous chondrocyte implantation (ACI) [11–13]. Several studies conclude that microfracture is not effective for larger lesions [14, 15]. The purpose of arthroscopic debridement is to remove loose intraarticular tissue debris and inflammatory mediators down to the subchondral bone, but not through it [13]. ACI attempts to re-implant the patient’s own chondrocytes over the defect to permit the chondrocytes to heal back onto the bone and mature into a hyaline-like cartilage. ACI have been directly compared to microfracture and mosaicplasty in randomized controlled trials [16–18]. The results indicate improved knee function in (carefully selected) patients [19], but studies have been criticized for methodological weaknesses [20]. The Cochrane database reported that there is insufficient evidence to conclude that ACI is superior to other treatment strategies for treating full thickness cartilage defect of the knee [21].

No statistically significant differences have been found between the different surgical treatments. This supports the suggestion that the improvement might be a result of the post-operative rehabilitation rather than the surgery itself [22, 23]. Furthermore, inclusion in a clinical trial is known to improve symptoms by itself, known as the Hawthorne effect [24].

All studies on cartilage reconstructive surgery have put the patients through a strict, careful, intensive and prolonged rehabilitation following the procedure, which could contribute to the clinical improvement observed. Wondrasch and co-workers implemented an active rehabilitation program in 48 patients with focal cartilage damage to a weight-bearing area of the knee [23]. None of the patients had any form of surgery to their injury. After three months there was a statistical significant and clinical meaningful improvement in KOOS score, load progression and hop score, to the point where 31 (65 %) of the patients declined further surgery for their cartilage lesion. This indicates that good results can be achieve with physiotherapy alone. Dozin and coworkers compared ACI versus mosaicplasty, where all candidates where evaluated arthroscopically with debridement of the lesion 6 months prior to definitive treatment [16]. All candidates completed an intensive rehabilitation protocol following the debridement, but before the cartilage surgery. This included non-weight bearing for two weeks and immediate active and passive physiotherapy. After two weeks isometric and proprioception exercises were introduced, as well as gradually strengthening exercises. 31 % of the candidates experienced substantial clinical improvement following the debridement and physiotherapy alone, and needed no further (surgical) treatment, questioning the need for cartilage treatment as a first port of call in such patients. The authors conclude that further randomized clinical trials are needed, where ACI should be compared to debridement alone. This is a missing link in our knowledge of cartilage reconstructive surgery and hampers further progression in cell therapy [16, 25].

### Purpose of this study

The purpose of this trial is to compare autologous chondrocyte implantation (ACI) with arthroscopic debridement (AD) in symptomatic cartilage injuries larger than 2 cm^2^ in patients aged 18–50 years old in regard to both subjective and objective variables at predefined times as described under “variables” in section 6.

## Methods/Design

The study is part of “The Norwegian Cartilage Project (NCP): A multidisciplinary Approach to Improve the Treatment of Injured Articular Cartilage”, which is a total of five studies regarding cartilage injuries in the knee, including clinical trials, register studies and basic research studies.

This study is a prospective, randomized, controlled study with 2 treatment arms (ACI and AD). Because one treatment arm consists of a two stage surgery, with a mini-open arthrotomy, while the other treatment arm is a single stage arthroscopy, it will not be possible to blind the patients as to what treatment they have received.

Inclusion and treatment will take place at Akershus University Hospital (Ahus) or Oslo University Hospital (Ullevål). The follow up appointments will be performed at Ahus or at the local NCP affiliated hospital by an external reviewer connected to the NCP group. The follow up will be blinded so that the examinator is unaware of which treatment arm the patient belongs to. To achieve this, the patient is instructed not to reveal the nature of the treatment he/she has received, and an opaque elastic stocking is put over the knee to conceal the scars (which are different between the treatments). Follow up is planned to 2 years, but each participant will also be invited to clinical and radiological follow ups after 5 and 10 years. The study has received ethical approval from the Regional Committee for Medical and Health Research Ethics North-Norway (Approval number 2015/2200). Written informed consent will be obtained from the subjects.

### Participants

The study will include a total of 82 patients, both men and non-pregnant women, with a full thickness or osteochondral defect in the weight bearing area of the femoral condyles or trochlea larger than 2 cm^2^. The lesion must be symptomatic, with a Lysholm score less than 75 [26]. The inclusion criteria are based on the recommendations by Brittberg [27], and include patients aged 18 to 50 years old with a stable knee, good range of motion (ROM), normal alignment (less than 5° varus or valgus measured on hip-knee-ankle (HKA) angle images) and no sign of radiologically osteoarthritis classified after Kellgren-Lawrence [28]. The weight-bearing, fixed-flexion posteroanterior radiographs will be obtained with the SynaFlex X-ray positioning frame (Synarc) [29].

### Inclusion

All patients will be assessed clinically as well as radiologically prior to inclusion to avoid peroperative exclusions. The full thickness or osteochondral defect will be verified arthroscopically as a grade 3 or 4 lesion according to the International Cartilage Repair Society (ICRS) [30]. Some patients might be excluded peroperatively based on arthroscopic findings such as osteoarthritis, less than 50 % normal meniscus or inappropriate size of the lesion [31]. The definitive inclusion of the patient will be performed by the operating surgeon during the diagnostic arthroscopy, prior to randomization. Patients who will be excluded based on peroperative findings will receive appropriate treatment according to the standard of care at the local hospitals.

Exclusion criteria include general osteoarthritis, systemic arthritis, severe co-morbidities that may influence surgery or rehabilitation potential, significant alcohol or drug abuse, psychiatric disorders, language barriers, pregnancy, severe obesity (body mass index > 30) and previous surgery to the chondral defect such microfracture or mosaicplasty (previous cruciate ligament reconstruction, fixation of osteochondritis dissecans lesions, alignment procedures (osteotomies) or meniscal surgeries are not exclusion criteria). Patients declining participation in the trial (and who fulfill the inclusion criteria) or withdrawing underway will receive appropriate treatment according to the standard of care at the local hospital.

The inclusion and exclusion criteria are presented in Table 1.

**Table 1:** Inclusion and exclusion criteria

Inclusion criteria	Exclusion criteria
-Age 18–50 years old	-Osteoarthritis, rheumatoid or other systemic arthritis
-Single symptomatic cartilage defect on medial or lateral femoral condyle or trochlea	-Malalignment >5° measured on HKA images
-Defect size larger than 2 cm^2^	-No radiological osteoarthritis
-Lesion graded ICRS 3–4	-Obesity (Body mass index > 30)
- > 50 % intact meniscus	-Comorbidities that may influence surgery or rehabilitation
-Ligamentous stable knee	-Pregnancy
-Acceptable range of motion (5–105°)	-Inability to complete questionnaires or rehabilitation
-Lysholm score < 75	-Serious alcohol or drug abuse
-Informed consent	-Previous surgery to the chondral defect except OCD surgery

### Randomization

The randomisation will be performed using a computer generator (randomization.org). 82 patient will be block randomized in pairs of six-eight (1:1) to treatment allocations. Each patient receives a patient number (1 through 82) on inclusion. Randomization will be printed in faded text and concealed in opaque numerically marked envelopes. The printing and concealing will be done by a person working at Akershus University Hospital, but is not involved in the study, to secure blinding. The randomization process occurs in the operation theatre during the arthroscopy after the operating surgeon has measured and graded the lesion to fulfill the inclusion criteria (see “Operative procedure”).

### Operative procedure

**Fig. 1:**
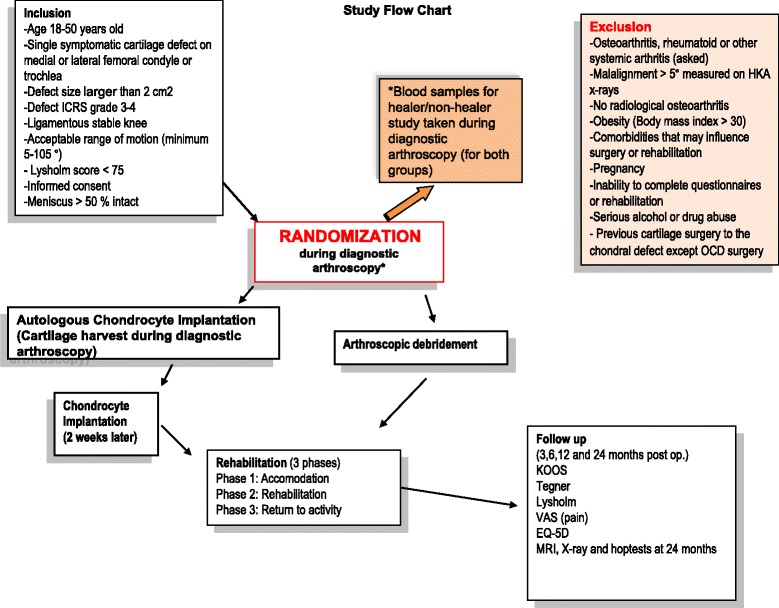
Study flow chart

In this study Autologous Chondrocyte Implantation (ACI) is compared with Arthroscopic Debridement (AD). All patients will undergo a diagnostic arthroscopy, and patients randomized to AD will have this procedure performed at the end of the diagnostic arthroscopy, while for the patients in the ACI group the chondrocyte implantation will take place two weeks later in a second stage operation as described below. The Study Flow Chart is presented in Fig. 1. The ACI technique is based on the technique described by Brittberg [32].

#### Diagnostic arthroscopy

Three standard incisions are made (supralateral for the patella and medial and lateral for the patellar tendon). A thorough diagnostic arthroscopic examination is then done. Removal of loose bodies and other necessary intraarticular procedures are done first (meniscus, plica). The focal cartilage lesion is then measured using a standard 4-mm arthroscopic probe and ICRS graded [30, 31].

Then either arthroscopic debridement (AD) or autologous chondrocyte implantation (ACI) is done, depending on randomization.

#### Autologous chondrocyte implantation

ACI is a three stage procedure taking place at Akershus University Hospital and Oslo University Hospital (Ex-Vivo Laboratory) over a two week period.

##### Stage 1: Debridement and cartilage harvest

Stage 1 includes a diagnostic arthroscopy with full inspection of the knee joint to ensure the inclusion criteria are fulfilled. Loose bodies are removed, any meniscal pathology is addressed. Inflamed synovium is debrided. The lesion is stabilized by debridement around the edges and down to the subchondral bone using a ring curette, but not through it, as described above. Cartilage biopsy is then taken from the non-weightbearing area of the medial aspect of the femoral notch. If this area does not contain enough healthy cartilage, the secondary donor site will be the non-weight bearing aspect of the lateral femoral condyle. Complications or donor site morbidity following arthroscopic harvesting of cartilage in this manner has not been described [27].

##### Stage 2: Chondrocyte growth in ex vivo laboratory Rikshospitalet, Oslo University Hospital

The harvested cartilage is transported to the cell culture laboratory in a sterile tube containing 0.9 % NaCl. The cells undergo mechanical mincing and antibiotic washing, and isolation of the chondrocytes by overnight collagenase digestion. The chondrocytes are then cultured for two weeks and resuspended to a treatment density of 30 million cells/ml. The cells are then transported back to the orthopaedic department on the day of the implantation.

##### Stage 3: Implantation of chondrocytes two weeks after initial arthroscopy

The chondrocyte implantation is performed under general or spinal anaesthesia, and a tourniquet inflated to 300 mmHg to achieve a blood-less field is applied to the upper thigh. A mini-open arthrotomy (medial or lateral depending of the location of the lesion) is performed and the lesion is assessed. The lesion is curetted down to subchondral bone, but care is taken to avoid bleeding. The surrounding cartilage is debrided to healthy tissue, exposing the lesion to bare bone. The lesion is measured and a template of sterile aluminium foil is used to model the exact shape of the lesion, overcorrecting with 1–2 mm.

The template is then used to cut out a matching piece of collagen sheet (ChondroGide® (Geistlich Pharma, Switzerland)) which is used to contain the cells in the defect. The flap is sutured to the lesion with 6.0 resorbable stitches and sealed with fibrin glue, leaving and opening at the upper part for injection of the cells. Saline is injected to the cavity to check for leakage, then aspirated before the cells are slowly injected using a soft catheter. The last opening is then closed with a last stitch and fibrin glue. The knee is then closed in the standard manner, taking care to close the capsule with subcutaneous resorbable sutures, before closing the skin incision with nylon sutures.

#### Arthroscopic debridement

The AD group will be subjected to a diagnostic arthroscopy with a full inspection of the knee joint to ensure the inclusion criteria are fulfilled. Lose bodies are removed, any meniscal pathology is addressed. Inflamed synovium is debrided. The lesion is stabilized by debridement around the edges and down to the subchondral bone using a ring curette, but not through it. Microfracture or any other cartilage treatment will not be performed.

No intra-articular local anesthetics will be used due to the possible harmful effect on cartilage [33–35]. All the operating surgeons will receive proper training in the operative procedure before study start.

### Post-operative management

The two treatment groups will receive identical postoperative care. The patients are usually admitted to the hospital for up to 4 days. No intravenous prophylactic antibiotics is given, anti-thrombotic prophylaxis is given only when there is a risk of thromboembolic disease (such as previous deep vein thrombosis or pulmonary embolus, protein C or S deficiency, Leiden V mutation and use of contraceptive pills). All patients will be given a sick leave up to 2 weeks after the operation.

If a patient experiences complications during treatment such as wound infections, catching or locking etc. the patient will receive medical attention and follow-up according to the problem.

### Rehabilitation protocol

The two treatment groups will receive identical rehabilitation protocol according to a modification of Wondrasch et al. [23]. The patients are admitted to the hospital for 2–4 days. The patients are seen by a physiotherapist and the surgeon day 1 after surgery to be instructed in range of motion exercises and restrictions according to Phase 1. The patient is seen within two weeks by a local physiotherapist who has received information about the rehabilitation program, and who will follow the patient through the program, supervised by the Project Physiotherapist.

The rehabilitation program is an active rehabilitation and education program divided into 3 phases: accommodation (1), rehabilitation (2) and return to activity (3) (Table 2). During the rehabilitation program the physical therapist focuses on explaining why the exercises are important and how the exercises should be performed and adjusted based on pain response and other symptoms. The rehabilitation program consists primarily of cardiovascular and knee/hip progressive resistance and neuromuscular training, including balance and plyometric exercises. The local physical therapist receives information about the rehabilitation program, including what kind of exercises the patients should perform.

**Table 2:** Rehabilitation protocol (identical for both groups)

Rehabilitation phases	Physiotherapy and activities	Objectives	Criteria for progression to next phase
Phase1: Accomodation	Education/coaching	Reduce pain and swelling	No pain & swelling during activities of daily living (ADL)
	Ice, elevation and compression	Normalize range of motion	Flexion 90°
	Isometric exercises	Regain quadriceps control	Normalized quadriceps activity while walking (clinical evaluation by the physical therapist)
	Range of motion		
	Gait training (no weight-bearing for two weeks)		
Phase2: Rehabilitation	Stationary bike cycling	Recovery of full range of motion	Full range of motion
	Progressive knee and hip resistance training	Normalize muscle strength	No pain or swelling during and after training sessions
	Neuromuscular training	Dynamic joint stability during ADL	Equally distributed weight on the lower limbs during weight-bearing exercises with no shift of the trunk (visually assessed by the physical therapist)
			Ability to stand on 1 limb on a flat surface for at lest 10 s
Phase 3: Return to activity	Knee and hip resistance training	Recovery of strength and neuromuscular control	Return to sport based on individual assessment
	Neuromuscular training	Return to activity/sport	
	Cardiovascular training		

To monitor the adherence to the rehabilitation program, all patients are asked to use training diaries to provide information about frequency, type of exercise, load progression and number of repetitions. In addition, the patients are asked to respond every second week to an online survey, with the questions; 1. Have you been to a supervised physical therapy session during the last two weeks?, 2. How many physical therapy sessions did you attend during the last two weeks? 3. What kind of training/exercises have you performed during the physical therapy sessions? and 4. What kind of activities have you performed during the last two weeks? All questions are followed by several predefined answers (closed answers), but also open answers are included to make comments if required. The online survey will continue as long as the patient is under the care of a physiotherapist, while the training diary will be continued to the end of the project (24 months) to estimate the amount of home exercise performed by the patient.

#### Phase 1 - Accomodation

Inpatient rehabilitation consists of placing the leg in a Continuous Passive Motion (CPM) machine within the range as tolerated due to pain and swelling, aiming to achieve 30–70° day 1 after surgery. The patients should use the machine for 6–8 h every 24 h. The physical therapist instructs the patients in exercises such as active dorsiflexion/ plantar flexion of the ankle to encourage lower extremity circulation and isometric contraction of the quadriceps, hamstrings, and gluteal musculature to maintain muscle tone and minimize muscle loss. This also includes abduction exercises for the hip. The patients remain non-weight bearing for two weeks, but are allowed touch-down weight-bearing (the foot or toes may touch the floor, but not support any weight) through the affected limb using crutches.

When discharged from the hospital, the patients are encouraged to continue range of motion exercises; flexion/extension of the knee 500 repetitions three times a day. In this phase, 2 to 3 supervised physical therapy sessions are scheduled for each patient. Interventions such as ice, compression, electrical muscle stimulation, muscle activation of the quadriceps, hamstrings, gastrocnemius and gluteal muscles and gait training is included. Swimming is allowed when the wounds are healed.

After two weeks, protected weight-bearing is carefully introduced within the pain threshold and gradually increased up to full weight-bearing (continues into phase 2). Crutches are used until the patient walks normal without limping.

#### Phase 2 - Rehabilitation

The patients attends 2 or more supervised physical therapy sessions per week, in addition they perform 1 to 2 unsupervised training sessions per week. Cardiovascular training on a stationary bike and progressive knee and hip resistance training and neuromuscular training are performed in this phase. All strengthening exercises are performed with both the injured and uninjured limbs. When full weight-bearing is achieved, long distance walking with increasing distances is encouraged and cross-country skiing can be allowed.

#### Phase 3 - Return to activity/sport

This phase is individualized according to the goals for each patient. The patients attend 1 or more supervised sessions per week, in addition they perform resistance training for a minimum of 2 and a maximum of 4 sessions per week. Cardiovascular and neuromuscular training can be performed daily. Where a return to sport is planned, it is important that sport-specific activities are included as functional progressions within the rehabilitation program.

### Outcome measures (dependent variables)

#### Demographics

Demographics to be collected at inclusion is age, gender, height (cm), weight (kg), BMI, injury mechanism (if any), previous medical history, current medication, smoking, social status, work status and nationality.

#### Primary endpoint

The Knee Injury and Osteoarthritis Outcome Score (KOOS) is a patient reported outcome measure validated to use in cartilage research studies and will enable comparison of our results with other reports. It assesses five domains; pain, symptoms, activity of daily living, sport and recreational function and knee-related quality of life [36]. KOOS knee-related quality of life (QoL) subscore is the primary endpoint, where the primary aim is the difference in KOOS QoL subscore in the ACI group compared to the AD group at 2 years follow up. There will not be any interim analysis before 2 years follow up.

#### Secondary endpoints

A combination of self-explanatory questionnaires, clinical parameters, clinical hop tests and radiographs and Magnetic Resonance Imaging (MRI) will be used as secondary endpoints. The secondary aims will be the difference between the two treatment groups and within the group at predefined times as described below. The secondary aims are:

- KOOS score: all subscores except the knee-related QoL subscore which is the primary aim.
- Tegner score; To evaluate the level of physical activity.
- Lysholm score; A condition-specific outcome score containing eight domains; limp, locking, pain, stair-climbing, use of support, instability, swelling and squatting.
- EQ-5D; A generic measure of health status that provides a simple descriptive profile used in the clinical evaluation of health care. EQ-5D is also widely used by clinical researchers and recommended for use in cost-effectiveness analyses by the Washington Panel on Cost Effectiveness in Health and Medicine [37] and by the International Society for Pharmacoeconomics and Outcomes Research (ISPOR) task force on good clinical practices: Randomized Clinical Trial-Cost-Effectiveness Analysis (RCT-CEA) [38].
- Visual analogue scale (VAS); A visual analogue scale for pain, where 0 represents no pain and 10 represents the worst pain imaginable.
- Range of motion (ROM) will be measured with a goniometer
- Costs; resource use related to the intervention, medication, rehabilitation, use of health care services and production loss.
- The patients will also provide information about work (return to work) physical activity and return to sport.

All outcome questionnaires will be completed by the patients before surgery (pre-operative or baseline values) and at the designated research follow-up appointments at 3 (±2 weeks), 6 (±4 weeks), 12 (±6 weeks), and 24 (±8 weeks) months.

At 24 months follow up, there will be 3 additional elements consisting of:

- Standing x-rays to evaluate any development of osteoarthritis.
- Magnetic Resonance Imaging (MRI); assessing the quality of the cartilage tissue, using a specific cartilage MRI technique, to assess the healing of the defect.
- A hop test, validated and described previously by Noyes, to assess clinical function [39].

We will also invite patients to attend a 5 year and 10 year follow up appointment. During these late controls, all the primary and secondary outcomes will be assessed for as described above.

### Hypothesis

Null hypothesis: There is no difference in KOOS subscore quality of life following ACI or AD treatment of a symptomatic cartilage defect (>2 cm^2^) in the weight bearing area of the knee 2 years after surgery.

The alternative hypothesis claims that differences between ACI and AD in KOOS quality of life subscore exist.

### Statistical analysis

Demographic and clinical characteristics will be presented as means and standard deviations (SD) or frequencies and percentages, as appropriate. The normality of continuous data will be assessed by examining the histograms. If necessary, a suitable transformation will be considered to symmetrize the data.

Due to along follow-up period with 5 (7) measurement points, repeated observations will be available for each patient. A mixed model correctly adjusting for intra-patient correlations will be used to assess the trend in primary and secondary end-points. The model will contain random effects for intercepts and slopes, if significant. Fixed effects for time (likely non-linear) and group will be included together with the interaction between the two. The interaction will quantify possible differences between arms regarding time profiles and serve as an omnibus test. For continuous end-points, linear mixed model will be estimated, while generalized linear model will be fitted to dichotomous end-points. Relevant pairwise comparisons will be performed by deriving individual time point contrasts within each study arm. The results will be presented as estimated means of odds ratios together with the corresponding 95 % confidence intervals (CI) and *p*-values. The estimates will further be adjusted for possible confounders such as age of the patient and severity of the cartilage lesion in the multivariate regression models.

The results with *p*-values below 0.05 will be considered statistically significant. Two-sided tests will be used. The data analysis will be conducted using SPSS v.22 (SPSS Inc, Chicago, Illinois) and SAS v 9.4.

### Sample size

It has previously been shown that a change in subscore of 8–10 of KOOS QoL (quality of life) is clinically significant [40, 41]. Therefore, for power analysis a difference in change of 10 between two treatment groups was assumed. A SD for change of 15 was used [42]. With the power of 80 % and significance level of 5 % the estimated minimum number of patients was 37 in each group. By adding 10 % due to loss during follow-up, we therefore plan to include a total of 82 patients.

### Risk assessment

Some patients may find it unpleasant when asked about demographic information. The treatment of choice in this patient population is not established, but standard clinical care include both arthroscopic debridement (AD) and autologous chondrocyte implantation (ACI). There are no additional risks of this study other than the potential risks of standard clinical practice.

Potential risk of AD is rare with a frequency < 1 % and include infection, deep vein thrombosis (DVT), arthralgia, headache, joint effusion/swelling, nasopharyngitis.

Potential risk of ACI is rare, but slightly higher than for AD, as this treatment involves a second operation with a mini-opening of the knee. Four major complications requiring surgery following ACI have been identified [43]:

- Malfusion of the repair tissue to the bed of the lesion (3.8 %)
- Delamination of healthy cartilage near lesion (2.8 %)
- Hyperthrophy of the repair tissue (>2 %)
- Insufficient degenerative tissue (>2 %)

Other complications are rare and occurs in < 1 % of patients, such as wound break down, infection, DVT and joint stiffness.

Both treatments have a risk of failure, meaning that the pain and stiffness in the knee does not improve despite surgery, and further surgery might be required.

## Discussion

The treatment of isolated full thickness cartilage lesions of the knee is difficult, and various surgical techniques are available. Since its introduction in the late 1980s Autologous Chondrocyte Implantation has become a recognize method for larger lesions. This method is costly and demands patience and strict compliance from the patients. The results have been promising, but the method has not been directly compared to physiotherapy. This study will therefore answer pertinent questions regarding the added benefit of ACI compared to physical rehabilitation alone. The results will help surgeons improve clinical outcome after cartilage injuries of the knee.

## Abbreviations

ACI: autologous chondrocyte implantation
AD: arthroscopic debridement
ADL: activity of daily living
AHUS: Akershus University Hospital
AOT: autologous osteochondral transplantation
BMI: body mass index
CI: confidence interval
CPM: Continuous Passive Motion
DVT: deep vein thrombosis
Eq5D: European quality of life five dimensions
HKA: hip knee ankle
ICRS: International Cartilage Repair Society
ISPOR: International Society for Pharmacoeconomics and Outcomes Research
KOOS: knee injury and osteoarthirtis outcome score
mmHg: millimeter of mercury
MRI: Magnetic Resonance Imaging
NaCl: sodium chloride
NCP: The Norwegian Cartilage Project
OD: osteochondritis dissicans
QoL: quality of life
RCT-CEA: Randomized Clinical Trial-Cost-Effectiveness Analysis
ROM: range of motion
SD: standard deviation
VAS: visual analogue scale
